# A Hybrid Multi-Criteria Decision-Making Approach Based on ANP-Entropy TOPSIS for Building Materials Supplier Selection

**DOI:** 10.3390/e23121597

**Published:** 2021-11-28

**Authors:** Chun-Ho Chen

**Affiliations:** Bachelor Program of Real Estate Investment and Management, Takming University of Science and Technology, No. 56, Sec. 1, Huanshan Rd., Neihu District, Taipei 11451, Taiwan; phdchen5598@takming.edu.tw

**Keywords:** decision-making approach, ANP-entropy weight, combination weighting method, TOPSIS

## Abstract

This article will tell you how to combine “entropy” in the model to reduce the bias of multi-criteria evaluation. Subjective weights are usually determined by decision makers based on their professional background, experience and knowledge, and other factors. The objective weight is obtained by constructing an evaluation matrix of the information based on the actual information of the evaluation criteria of the scheme, and obtained through multi-step calculations. Different decision-making methods are based on different weight types. Considering only one of the two weights often leads to biased results. In addition, in order to establish an effective supply chain, buyers must find suitable merchants among suppliers that provide quality products and/or services. Based on the above factors, it is difficult to choose a suitable alternative. The main contribution of this paper is to combine analytic network process (ANP), entropy weight and the technique for order preference by similarity to an ideal solution (TOPSIS) to construct a suitable multi-criteria decision (MCDM) model. By means of ANP-entropy weights to extend the TOPSIS method, ANP-entropy weights are used to replace subjective weights. A supplier selection decision-making model based on ANP-entropy TOPSIS is proposed. At last, the sensitivity analysis shows that, taking the selection of building materials suppliers as an example, the hybrid ANP-entropy TOPSIS method can effectively select suitable suppliers.

## 1. Introduction

The decision-making process usually needs to consider multiple attributes and criteria at the same time, and multiple technologies and methods are needed to assist decision making. In the field of multi-criteria decision making, decision makers should follow rational principles and logical estimations when deciding on the most appropriate plan, that is, weighting and evaluating interdependent or mutually independent limited criteria [1].

Supplier evaluation and selection is an important part of business operations. The main purpose of the evaluation process is to select the most suitable supplier, not to find the supplier with the best technology, the shortest delivery period, or the lowest price [2,3].

Supply chain management (SCM) is mainly to improve customer satisfaction, meet consumer service needs, and maximize revenue and profitability, reduce manufacturing costs, optimize business processes, cycles, and inventory levels to improve enterprise competitiveness [4,5,6,7,8,9]. In the early stages of the supply chain, selecting the most advantageous supplier is one of the most important tasks. In the decision-making method for the management of complex areas in the supply chain, it is especially necessary to determine certain criteria in advance [10]. In recent years, academia and practice have conducted extensive research on supply chain management.

One of the necessary conditions for the success of the supply chain is an effective procurement mechanism [4,11,12,13]. The correct selection of suppliers can save a huge cost for the company, and it is also an important responsibility of the procurement agent [14]. System analysis puts forward several methods for supplier suitability selection for discussion, including Analytic Hierarchy Process (AHP) [15,16], supplier performance matrix method [17], supplier profile analysis [18], matrix method [19], taxonomy and weighted point method [20,21].

The supplier selection problem has the characteristics of multiple indicators, unstructured, complexity, and diversity. This is a question of choice under multi-criteria conditions [22,23,24,25].

Subjective and objective factors are often not considered at the same time in decision-making tasks, such as failure to consider data information, incorrect expression of preferences, qualitative standards, etc. [26,27]. Most decision-making methods are discussed for solving supplier selection issues under non-complex circumstances [28]. The article proposes a fuzzy analytic hierarchy process structure model to construct a rubber supplier evaluation [14].

Hwang and Yoon proposed a sorting method based on the similarity with the ideal solution (TOPSIS), which is a commonly used multi-criteria decision-making method [29]. The reason for choosing the TOPSIS method is that TOPSIS is one of the known classic multi-attribute decision-making methods and has been widely used in many literatures. The TOPSIS method includes both a negative ideal solution of the cost type and a positive ideal solution of the benefit type. A suitable supplier selection should be far from the negative ideal solution and closer to the positive ideal solution.

The research method of TOPSIS can solve the problem of supplier selection objectively and effectively, so the academic circles attach great importance to it and regard it as one of the main research topics [30,31]. However, TOPSIS still has its shortcomings, the main reason is that the weight of TOPSIS must be subjectively determined by the decision maker [3,32]. At the end of the article, we use the TOPSIS method to sort all alternatives and select the most suitable alternative [33].

In order to effectively solve the problem of supplier selection, a hybrid decision-making approach based on ANP-entropy TOPSIS is proposed. Based on the in-depth analysis of the above information, the main research topics are proposed as follows:

(1) The TOPSIS method has disadvantages in the weight setting due to the subjective judgment of the decision maker. Therefore, when the decision-maker’s subjective consciousness is too strong or the information obtained is incomplete, how should it be solved?
(2) The entropy weight measurement of the first level of ANP is different from the entropy weight calculation of the second level. How to combine different entropy weights with the TOPSIS method to obtain objective weight values?
(3) The weight estimated by the entropy weight method is an objective weight, which makes up for the lack of subjective weight in the ANP method. Based on the two subjective and objective weights of ANP and the entropy method, how to obtain the ANP-entropy weight and combine it with TOPSIS?

In order to select suitable building material suppliers, in this research, we propose a hybrid multi-criteria decision-making model based on ANP-entropy TOPSIS. The ANP-entropy weighted TOPSIS method has a great opportunity for application and success in the process of supplier selection.

In addition to the introduction, the rest of this article is divided into five parts. The second section reviews comprehensive literature and research methods. The third section introduces the construction steps of ANP-entropy weighted TOPSIS. Section four shows examples of numerical execution selected by building material suppliers. Section five is the results and discussion. Finally, the sixth part gives the conclusion of this research.

## 2. Literature Review and Methodology

**Figure 1:**
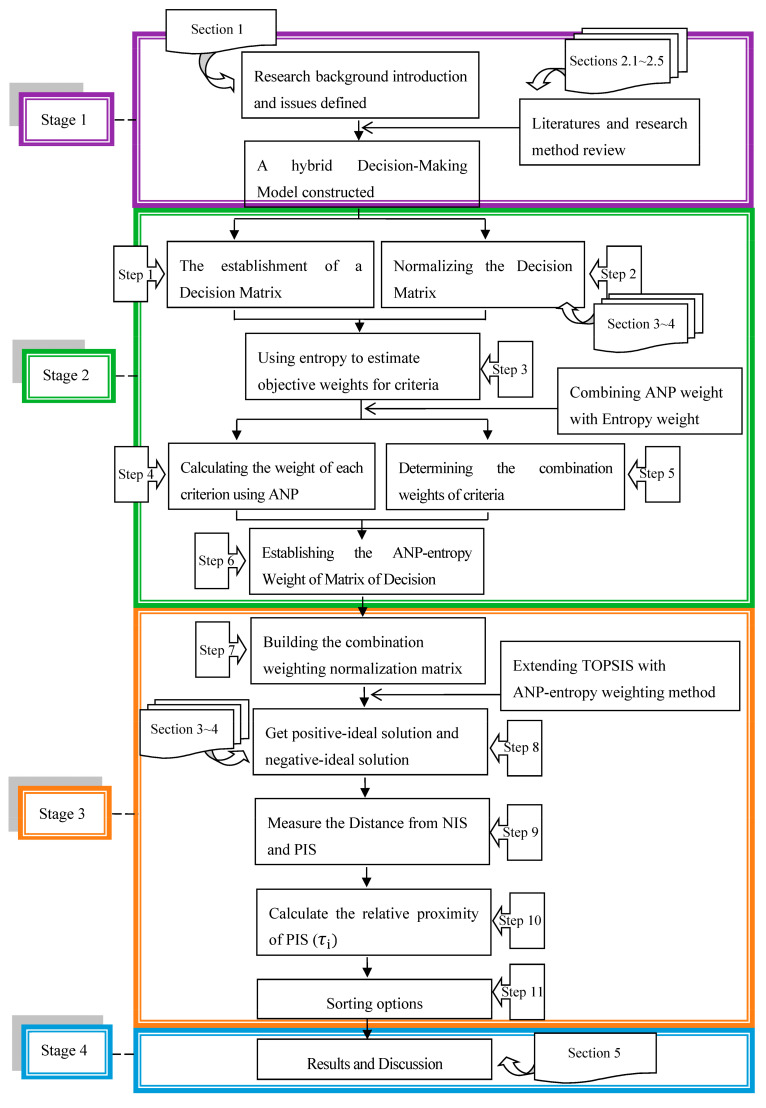
Research structure and analysis steps.

The research framework consists of four stages, and the analysis process is shown as Figure 1:

Stage 1: This article’s research background introduction, literature and research method review.

Stage 2: The construction of a new TOPSIS model, and then combined ANP weight and entropy weight.

Stage 3: Extending the TOPSIS model combined with the ANP-entropy weighting method.

Stage 4: Results and discussion.

The first stage introduced Section 2.1, Section 2.2, Section 2.3, Section 2.4 and Section 2.5, including methodologies, such as TOPSIS method, ANP method, entropy weight method, combined weighting method and other related literatures. As for Section 3, Section 4 and Section 5, they will be introduced at other stages. The sixth part is the conclusion of this research.

### 2.1. Literature Review

#### 2.1.1. Literature on the Application of the Entropy, ANP, Fuzzy, Grey, Neutrosophic, CILOS, IDOCRIW and/or TOPSIS Method

This section discusses the literature related to entropy, ANP, fuzzy, grey, neutrosophic, CILOS, IDOCRIW and/or TOPSIS method researched by researchers. Information entropy originates from information theory [34], which was initially used to evaluate the uncertainty of hydrological models [35]. The experimental results show that entropy information can significantly improve the robustness and recognition rate of the algorithm [36]. A research is based on TOPSIS technology and uses entropy weight information to calculate the weight of the criteria, in order to selecting suitable suppliers in a green environment [37]. Using the TOPSIS method and the entropy weight method, a simulation-based multi-objective evaluation model for water flow corridors is established [38].

Because ANP considers the complex and interrelated relationships between decision-making elements and can apply qualitative and quantitative attributes, it is widely used to solve practical problems [39]. The Fuzzy Analysis Network Process (F-ANP) is used to evaluate the weight of the standard, and the fuzzy symmetry technique is used to determine the impact of the alternative through the similarity to the ideal solution (TOPSIS) [40]. The Analytical Network Process (ANP) is used to calculate the weight of selected criteria by considering their interdependencies. In order to avoid additional comparisons of the analysis network process, the TOPSIS method is used to rank the alternatives [41]. Based on the Fuzzy Delphi Method, DEMATEL, Analytical Network Process (ANP), and TOPSIS, a hybrid multi-criteria decision-making (MCDM) model to select the best variety show for TV stations in the social media era show host is established [42].

To select the most suitable location for Indian thermal power plants based on the social, technological, economic, environmental, and political (STEEP) fuzzy AHP-TOPSIS framework. Using the fuzzy analytic hierarchy process to determine the weights of qualitative and quantitative indicators that affect the site selection process [43]. Combine AHP and TOPSIS methods with neutral (N) theory to deal with complexity, uncertainty, and ambiguity [44].

Combining the grey comprehensive evaluation method (GCE) with the TOPSIS method, a new hybrid multi-scale decision-making method—the grey fuzzy TOPSIS method (FGT) is proposed to improve its one-sided problem [45].

Neutrosophic sets is a method to solve problems combined with AHP, TOPSIS and other technologies in recent years [46]. Neutrosophic sets use three membership functions that express accuracy, inaccuracy, and uncertainty, and it has been recognized as an effective method for solving complex decision-making (DM) problems. The study considers a new extension of the TOPSIS method applicable to single-valued neutrosophic sets [47].

Develop a new method of group decision making (GDM) in an intuitionistic fuzzy environment to help managers make more accurate decisions [48].

Compare the ranking results of two MCDM methods (ie, analytic hierarchy process (AHP) and network analysis (ANP)) combined with the ideal solution similarity ranking technology (TOPSIS), and use the Shannon entropy method to calculate the objective weight for each criterion. These weights are combined with TOPSIS to obtain an objective ranking of alternatives [49].

An extended cloud TODIM (Tomada de Decisão Iterativa Multicritério) method is proposed, which describes the evaluation information through a credible fuzzy language term set (HFLTS), and converts the fuzzy language term set into a cloud to fully describe the ambiguity, uncertainty, and randomness. Then, a combination of network analysis method (ANP) and entropy weight method is used to calculate the criterion weight [50].

By reconstructing the analytical network process (ANP) and entropy weight method (EWM), a new hybrid multi-criteria decision (MCDM) method for offshore wind turbine selection is designed. Based on the weights assigned to ANP and EWM, the best alternative can be selected [51].

This research revises the traditional performance analysis (IPA) method, and uses the comprehensive weight-(ANP-) entropy weight method obtained by analyzing the network process to obtain the importance of the project [52].

Considering the information utility and interaction of indicators, a comprehensive weighting method based on standard deviation is proposed. The results show that the entropy weight method and the improved ANP algorithm have good consistency and significant correlation, and the comprehensive weight method is effective and reliable [53].

Establish a cloud-TODIM framework to deal with the problem, apply the hesitant fuzzy language term set (HFLTS) and cloud model to describe uncertain information, combine the analytical network process (ANP) method and the entropy method to obtain the criterion weight, which can avoid the weight determination too subjective, and you can measure the mutual influence between the standards at the same time [54].

These criteria weights are determined by the combined ANP-entropy method. In addition, considering the psychological characteristics of decision makers, the TODIM (Portuguese acronym for Interactive Multi-criteria Decision Making) method is used to rank the overall risk level of CFPP investment in 23 countries [55].

According to the similarities with multi-criteria decision making (MCDM), the ideal solution (TOPSIS), Shannon entropy (SE), and the analytical network method (ANP) methods, priority is given to the implementation of strategic plans, such as economic conditions, managerial opinions, consensus, city council approvals, and national documents [56].

Combining subjective (analysis of the network process) and objective (entropy weight method) evaluations, comparing China’s policies in three dimensions: environment, energy, and economy with traditional energy policy evaluation research, makes the evaluation results more reasonable and reliable [57].

This research proposes an influencing factor system based on the combination of entropy and the subjective and objective weights of the analytical network process (ANP), which avoids the complicated optimization process. It is a new attempt to optimize the production plan based on the fuzzy comprehensive evaluation of ANP, which enriches the process optimization methods in manufacturing to a certain extent [58].

A multi-attribute decision-making method based on combined weights and GI-TOPSIS is proposed, which combines ANP and entropy methods to consider the dependence of evaluation indicators and the information of evaluation data, and introduces gray-level correlation into TOPSIS. A multi-attribute decision-making method based on combined weights, GI-TOPSIS, is proposed, which combines ANP and entropy methods to consider the dependence of evaluation indicators and the information of evaluation data, and introduces gray-level correlation into TOPSIS [59].

Entropy methods are widely used to determine the weight (importance) of the criteria. When choosing another criterion as the best standard, a new criterion impact loss method, CILOS, is used to determine the relative impact loss experienced by the criterion of an alternative. The author of this paper combines the best features of the entropy method and the CILOS method to obtain a new method- Integrated Determination of Objective Criteria Weights, or (IDOCRIW) [60].

The FIDOCRIW method proposed in this paper retains the idea of combining entropy and CILOS methods in the IDOCRIW method. In contrast, FIDOCRIW deals with fuzzy numbers instead of real numbers. This method includes data uncertainty and allows the fuzzy structure of the decision matrix to be completely retained along the entire framework of the method [61].

A new interval entropy method is proposed for the recursive process of sorting alternatives. Compare three alternative methods based on entropy applied to solve the MADM problems-entropy method for determining the criterion weight (EMDCW), method of criteria impact LOSs and determination of objective weights (CILOS) and integrated determination of objective criteria weights (IDOCRIW)[62]. Applying the TOPSIS and ELECTRE-I model to medical diagnosis fuzzy information [63]. The Delphi-DEMATEL-ANP-TOPSIS hybrid model was established, and the strategy for handling the train derailment risk was selected [64].

On this basis, research methods for solving problems and constructing models are proposed.

#### 2.1.2. Rank Reversals in Decision-Making

Rank reversal refers to a ranking change in a multi-criteria decision-making process. The ranking change will overwrite the originally possible decision sequence when, for example, selecting a set of other alternative projects or changing the method. In multi-criteria decision making and many decisions, the issue of ranking reversal is at the core of many debates. The research results prove that the multi-criteria decision-making method may have various types of ranking reversals, such as [65,66,67,68,69,70]:

1. The TOPSIS method.
2. The PROMETHEE (outranking) method.
3. The analytic hierarchy process (AHP) and some of its variants.
4. Multi-attribute utility theory (MAUT).
5. The ELECTRE (outranking) method and its variants. Since ranking reversal exists in the above multi-criteria decision-making model, the mixed multi-criteria decision-making model may also have limitations. For example, the decision model is a hybrid model of ANP and TOPSIS.

### 2.2. ANP Method

#### 2.2.1. The Meaning of ANP

In the real situation, there is often mutual dependence between the upper and lower levels and a net-like relationship of mutual interaction, rather than a purely linear relationship from top to bottom. Analytic Network Process (ANP) is a decision-making method that adapts to the non-independent “hierarchical structure” proposed by Professor Satty of the University of Pittsburgh in 1996. The ANP method is a generalization of AHP, mainly adding a feedback mechanism to AHP [71]. The main difference between the ANP method and the AHP method is that the ANP rule is applied to related problems where the schemes or criteria are mutually dependent, while the AHP method is only used to solve the related problems when the schemes or criteria are mutually independent. The level difference between the two is shown in Table 1.

**Figure 2:**
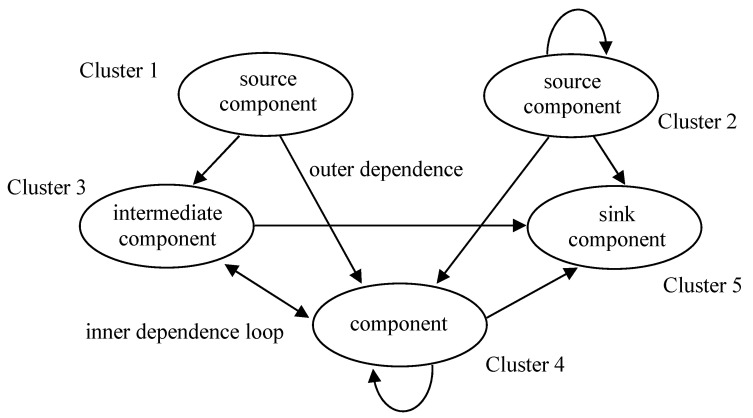
Network diagram of dependent system among groups or components in ANP method [56].

The ANP method contains two kinds of dependencies, namely, the internal dependence between the elements in the same group, and the external dependence between the group and the group organization. Satty believes that the interdependent interaction relationship between groups and elements can be presented graphically, as shown in Figure 2.

**Figure 3:**
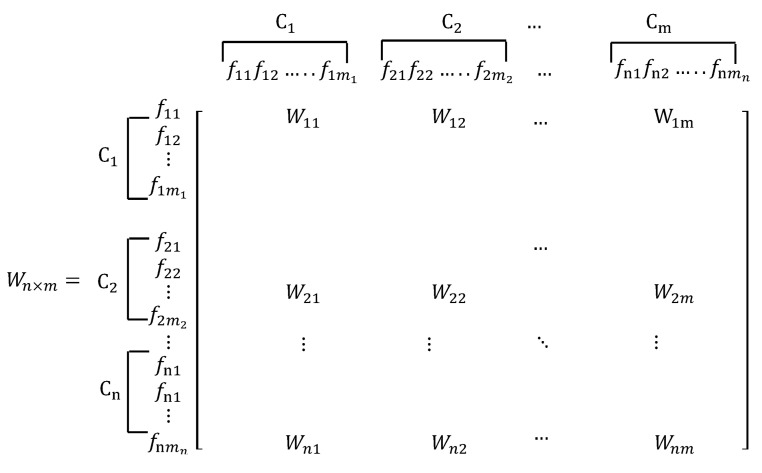
Supermatrix [54].

In addition, the ANP method uses a supermatrix to express the relationship and intensity between the elements in the graph, as shown in Figure 3.

Among them, *C*_*i*_ represents a decision criterion, $f_{i1}$, $f_{i2}$, $f_{im_{i}}$ indicates the evaluation element under the *i*-th criterion, I = 1, 2,…, n. $W_{n1}$ $W_{n2}$, …,$\;W_{nn}$ are the resulting eigenvector values. In the calculation and analysis process, the priority ratio between the elements in the hierarchical structure is represented by eigenvectors, and then the eigenvalues are calculated as the basis for evaluating the consistency of the dual comparison matrix. If the consistency conditions are met, the priority order represented by the eigenvector can be used as the basis for decision making or selection [73,74,75,76].

#### 2.2.2. Application of ANP

Analytic Network Process (ANP) is a well-known pairwise comparison technique in multi-criteria decision models [77]. ANP is derived from a representative method of basic MCDA, the Analytic Hierarchy Process (AHP). It uses network mode instead of unidirectional hierarchical structure [71]. It can clarify the interdependence in the evaluation component groups (dimensions or standards) ignored by the traditional hierarchical structure [78]. In order to effectively explain the interdependence in ranking evaluation, Saaty [71] used the pairwise comparison operation of the AHP expert group. After the consistency check, the evaluation value of the eigenvector (EV) is obtained to construct the supermatrix. Therefore, the weight value of the constituent elements is calculated to be used in the evaluation decision. The construction of the supermatrix is similar to the concept of “row is equal to 1 randomly” in the Markov chain [79].

Due to the above characteristics and objective mathematical calculations, no matter how small the difference in weight levels, any ANP element that appears in the overall evaluation structure is considered to be directly related to the overall evaluation. Therefore, the evaluation ranking results can be presented objectively [80,81]. Based on the use of comparative factors and sub-factors, and the possible interdependence between them, this technology has several advantages [82].

#### 2.2.3. Steps of ANP Method

The operation of the ANP method is similar to that of the AHP method and can be divided into the following stages [80]:

1. Problem proposal and structure establishment

**Figure 4:**
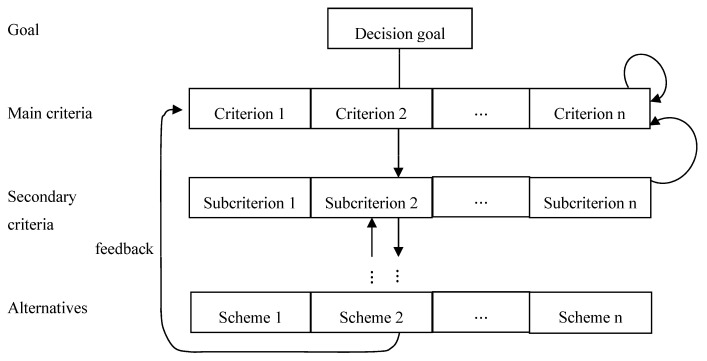
Problem structure schematic diagram of the ANP method.

First, we must clearly describe the problem and break it down into a networked hierarchical structure, as shown in Figure 4.

2. Build a pairwise comparison matrix

Pairwise comparison can be divided into two parts, one is the pairwise comparison of groups, and the other is the pairwise comparison of elements. In addition, further calculate the consistency of the results of the pair comparison matrix to determine whether the decision makers are consistent when they make pair comparisons.

3. Calculate the relative weight of each matrix

The eigenvectors calculated after the pairwise comparison of each matrix are used as the weight value of the matrix, and the value of each matrix is calculated one by one. Then, fill in the dependency table according to the interdependence between the elements to form an unweighted super matrix.

4. Calculate the combined weight of each horizontal (level) element

Obviously $\sum_{j=1}^{n}q1_{j}=1$, and according to Table 2, the combined weight of each horizontal (level) element can be calculated.

5. Form a super matrix

After multiplying the unweighted supermatrix multiple times, a stable convergence value that does not change is obtained, which is the limiting supermatrix.

6. Choose the best solution

Decision makers can use the extreme value obtained by multiplying the supermatrix multiple times as the basis for choosing the best solution.

### 2.3. Entropy Weighted Method

#### 2.3.1. Entropy Weight Principle

The entropy concept is what the German physicist R. Clausius proposed in 1865. It is the state parameter of matter, which describes the chaos or disorder or chaos of the thermodynamic system. Information entropy is used to measure the uncertainty of the signal in the information source, introduced by Shannon in 1948.

The entropy method calculates the relative weight between attributes and calculates the ability of each evaluation attribute to transmit decision information. It mainly uses the entropy value in information theory to express the uncertainty of information. Entropy weight can be calculated according to the judgment matrix [75,83,84,85]. “The greater the weight of the information criterion, the smaller the entropy of the evaluated information criterion”. 

#### 2.3.2. Significance and Nature of Entropy Weight Method

The entropy weight method uses the difference of indicators to measure the effective information contained in the known data and the weight of the indicators, which is to calculate the information entropy of the indicators. Entropy weight is not the index importance coefficient in the actual sense, but the relative importance coefficient of each index in competition when making a decision or evaluation plan under the conditions of a given evaluation object and evaluation index. Its characteristics are as follows:

- An indicator if the data of each evaluation object is the same, the indicator does not contain any valuable information. If the values of the elements in the column are the same, the entropy weight is 0, and the maximum entropy is 1.
- The greater the difference between the values of a column of elements, the larger the entropy weight of the column of elements, the smaller the entropy value, which indicates that the indicator has valuable information. Conversely, if the entropy weight of an indicator is smaller and the entropy value is larger, the importance of the indicator is smaller.

The calculation steps of entropy weight are as follows:

1. Normalization of the initial data matrix

In the initial data matrix of the entropy evaluation system, m evaluation objects and n evaluation indicators will be set.

(1) $$Y=\left[\begin{array}{cccc} y_{11} & y_{12} & \ldots & y_{1n}\\ y_{21} & y_{22} & \ldots & y_{2n}\\ \vdots \; & \vdots & \;\vdots \\ y_{m1} & y_{m2} & \ldots & y_{mn} \end{array}\right]=(Y_{1\;}Y_{2\;}\ldots \;Y_{n\;})$$

where $y_{\mathrm{ij}}\;$(i=1, 2, …, m;j=1, 2,…, n) denotes the value of the i-th evaluation plan in the j-th index, and *Y*_j_ (j = 1, 2,…, n) represents the column vector data of all evaluation schemes of the j-th index.

Since the indicators may have different units, each indicator needs to be standardized to eliminate the influence of different units on the evaluation results. The commonly used method is the step transformation method, and its calculation formula is:

(2) $$Y_{\mathrm{ij}}^{\prime }=\frac{\underset{i}{\max}\left\{y_{\mathrm{ij}}\right\}-y_{\mathrm{ij}}}{\underset{i}{\max}\left\{y_{\mathrm{ij}}\right\}-\underset{i}{\min}\left\{y_{\mathrm{ij}}\right\}}\;\mathrm{or}\;Y_{j}^{\prime }=\frac{\underset{i}{\max}\left\{y_{\mathrm{ij}}\right\}-y_{\mathrm{ij}}}{\underset{i}{\max}\left\{Y_{j}\right\}-\underset{i}{\min}\left\{Y_{j}\right\}}\;(\mathrm{applicable}\;\mathrm{cost}\;\mathrm{indicators})$$

(3) $$Y_{\mathrm{ij}}^{\prime }=\frac{y_{\mathrm{ij}-}\underset{i}{\min}\left\{y_{\mathrm{ij}}\right\}}{\underset{i}{\max}\left\{y_{\mathrm{ij}}\right\}-\underset{i}{\min}\left\{y_{\mathrm{ij}}\right\}}\;\mathrm{or}\;Y_{j}^{\prime }=\frac{y_{\mathrm{ij}-}\underset{i}{\min}\left\{y_{\mathrm{ij}}\right\}}{\underset{i}{\max}\left\{Y_{j}\right\}-\underset{i}{\min}\left\{Y_{j}\right\}}\;(\mathrm{applicable}\;\mathrm{benefit}\;\mathrm{indicators})$$

2. Estimate the proportion of the j-th index, and the i-th evaluation plan $y_{\mathrm{ij}}^{\prime }$

(4) $$q_{\mathrm{ij}}=\frac{y_{\mathrm{ij}}^{\prime }}{\sum_{i=1}^{m}y_{\mathrm{ij}}^{\prime }}\;\mathrm{or}\;Q_{j}=\frac{y_{j}^{\prime }}{\sum y_{j}^{\prime }}\;(j=1,\;2,\ldots ,\;n)$$

Therefore, the weight matrix can be calculated as:

(5) $$Q={\left(q_{\mathrm{ij}}\right)}_{m\times n}.\;\mathrm{or}\;Q=(Q_{1\;}Q_{2\;}\ldots {\;Q}_{n\;})$$

3. Calculate the information entropy value of the j-th indicator $e_{j}$

(6) $$e_{j}=-\pi \sum_{i=1}^{m}q_{\mathrm{ij}}\ln q_{\mathrm{ij}}\;(j=1,\;2,\ldots ,\;n)$$

where , 0 ≤ e_j_ ≤ 1, and $\pi =\frac{1}{\ln m}$ is non-negative constant. When set $q_{\mathrm{ij}}=0$, $q_{\mathrm{ij}}\ln q_{\mathrm{ij}}$ = 0.

4. Calculate the information utility t_j_ for the j-th indicator

(7) $$t_{j}=1-e_{j}\;(j=1,\;2,\ldots ,\;n)$$

5. Calculate the weight $w_{j}\;$of the j-th indicator

(8) $$w_{\mathrm{ej}}=\frac{t_{j}}{\sum_{j=1}^{n}t_{j}}=\frac{1-e_{j}}{n-\sum_{j=1}^{n}e_{j}}\;(j=1,\;2,\ldots ,\;n)$$

6. Estimate the evaluation value $U_{i}$ of the evaluation plan i

(9) $$U_{i}=\sum_{j=1}^{n}q_{ij}w_{ej}\;(i=1,\;2,\ldots ,\;m)$$

The greater the dispersion of the index, the smaller the entropy value. The results show that the greater the impact of the index on the comprehensive evaluation index, the greater the usefulness of the information.

### 2.4. Combination Weighting Method

The method in which objective weights and subjective weights are comprehensively considered and evaluated is the combined weight method. The weight value of the criterion has an important influence on the choice of the scheme in the multi-criteria decision-making evaluation method because it can affect the evaluation result. When the combined weight method is applied to the selection plan, it can reduce the deviation that may be caused by a single objective or subjective weight [1]. Assuming that the number of evaluation index items is n at a certain facet or in a certain evaluation level.

The weights determined by the entropy weight method and the ANP method are${\;W}_{e}=\left(w_{e1},w_{e2},\ldots ,w_{\mathrm{en}}\right)$ and $W_{p}=\left(w_{p1},w_{p2},\ldots ,w_{\mathrm{pn}}\right)$. Combining the weight values of the objective weight and subjective weight of the n criteria, the combined weight value is calculated as follows:

(10) $$w_{bj}=\frac{w_{pj\times }w_{ej}}{\sum_{j=1}^{n}w_{pj}\times w_{ej}}\;,\;j=1,\;2,\ldots ,\;n$$

### 2.5. Weights for Multi-Criteria Decision Making

The evaluation of criterion weight has a great influence on the choice of scheme in multi-criteria decision making. In other words, different evaluation results may result from different criterion weights. In principle, the criterion weight can be divided into the following three types of calculation methods:

1. Objective weight:

The objective weight is calculated based on the evaluation matrix. The calculation methods include: (1) the matrix method of grey relation [86]; (2) the entropy weight method [29]. The application of objective weights is limited to the evaluation of quantitative criteria and the source of quantitative data should be trustworthy. This basic assumption is important and necessary.

2. Subjective weight:

Subjective weight mainly comes from the subjective consciousness of decision makers or experts. In addition, many studies have also explored other methods, such as: (1) the AHP method [87]; (2) the ANP method [71]; (3) the weighted least square method [88]; (4) the extreme weight approach [89]; (5) the linear programming techniques for multidimensional analysis of preference (LINMAP) method [90].

Subjective weighting can be applied to the evaluation of quantitative and qualitative standards, especially when the background knowledge of experts is needed.

3. Eclectic weights:

The calculation of the eclectic weight adopts the combined weighting method. The purpose of the eclectic weight is to calculate the weight of each criterion while simultaneously considering the subjective weight of the decision maker and the objective weight calculated by the entropy method. Its advantages include the following [91]:

(1) Highly reliable analysis results;
(2) Reduction of deviation of evaluation results;
(3) The deviation between subjective weight and objective weight can be balanced and compromised.

## 3. Construction Steps of ANP-Entropy TOPSIS

Denote the set of criteria as R = {${\;R}_{1},R_{2},\ldots ,R_{n}$}, and the set of alternatives as L = {$L_{1},L_{2},\ldots ,L_{m}$}. The construction process of the ANP-entropy TOPSIS method is as follows:

Step 1: Establishing a decision matrix.

From the first step, we get the performance value and establish a decision matrix C = ${\left[c_{\mathrm{ij}}\right]}_{m\times n}$. The following shows the evaluated decision matrix:

(11) $$C=\begin{array}{cc} & \begin{array}{cccc} \;R_{1}\; & R_{2}\; & \cdots \; & R_{n} \end{array}\\ \begin{array}{c} L_{1}\\ L_{2}\\ \vdots \\ L_{m} \end{array} & \left[\begin{array}{cccc} C_{11} & C_{12} & \cdots & C_{1n}\\ C_{21} & C_{22} & \cdots & C_{2n}\\ \vdots & \vdots & \ddots & \vdots \\ C_{m1} & C_{m2} & \cdots & C_{mn} \end{array}\right] \end{array}$$

The decision matrix C contains m alternatives and n criteria. In addition, L stands for alternatives and R denotes the criteria.

Step 2: Standardizing the decision matrix.

In order to make the performance evaluation have a consistent measurement unit, the statistical normalization method is used to normalize the performance value. The performance value ($V_{\mathrm{ij}}$) after the normalization process is expressed as follows

(12) $$V_{\mathrm{ij}}=\frac{c_{\mathrm{ij}}}{\sum_{i}c_{\mathrm{ij}}},\forall i,\;j$$

The newly constructed decision matrix can be expressed as:

(13) $$C^{*}=\begin{array}{cc} & \begin{array}{cccc} \;R_{1}\; & R_{2}\; & \cdots \; & R_{n} \end{array}\\ \begin{array}{c} L_{1}\\ L_{2}\\ \vdots \\ L_{m} \end{array} & \left[\begin{array}{cccc} V_{11} & V_{12} & \cdots & V_{1n}\\ V_{21} & V_{22} & \cdots & V_{2n}\\ \vdots & \vdots & \ddots & \vdots \\ V_{m1} & V_{m2} & \cdots & V_{mn} \end{array}\right] \end{array}$$

Step 3: Using the entropy method to calculate the objective weight of the criterion.

The information entropy value ($e_{j}$) of the j-th criterion can be expressed as follows:

(14) $$e_{j}=-\pi \sum_{i=1}^{m}q_{\mathrm{ij}}\ln q_{\mathrm{ij}}.\;(j=1,\;2,\ldots ,\;n)\;$$

where $q_{\mathrm{ij}}=\frac{v_{\mathrm{ij}}}{\sum_{i=1}^{m}v_{\mathrm{ij}}}$, $\pi =\frac{1}{\ln m}$ is a non-negative constant. After that, calculating the weight W_ej_ of the j-th index.

(15) $$w_{ej}=\frac{1-e_{j}}{n-\sum_{j=1}^{n}e_{j}}\left(j1,2\ldots ,n\right)$$

Step 4: Calculating the criteria weights with the ANP method.

According to the steps of the Section 2.2.3 ANP method, the ANP weight of each criterion can be obtained in the following ways

(16) $$w_{pj}=\left(w_{p1},w_{p2},\ldots ,w_{pn}\right)\;(j=1,\;2,\ldots ,\;n)$$

Step 5: Calculating the combined weight of each criterion.

According to Equations (15) and (16), on the basis of the combined weighting method, the weight of each criterion can be determined as shown below:

(17) wibj=wipj×wiej∑j=1nwipj×wiej , i=1, 2,…, m; j=1, 2,…, n

where $w_{pj}$ is the subjective weight derived from the ANP method and $w_{ej}$ is the objective weight derived from entropy.

Step 6: Building the decision matrix based on the ANP entropy weight.

The ANP-entropy weight of the decision matrix can be shown as follows:

(18) wbj=w1bi×w2bj , i=1, 2,…, m; j=1, 2,…, n

Step 7: Establishing the normalized decision matrix on the basis of combined weights.

In order to show the relationship between weight and performance value, that is, the greater the weight of the evaluation standard, the more important its performance value. The performance value of the criterion must be reflected by multiplying the weights together. The combined weighted normalized decis decision matrix can be shown as:

(19) $$Z=\begin{array}{cc} & \begin{array}{cccc} \;R_{1}\; & R_{2}\; & \cdots \; & R_{n} \end{array}\\ \begin{array}{c} L_{1}\\ L_{2}\\ \vdots \\ L_{m} \end{array} & \left[\begin{array}{cccc} Z_{11} & Z_{12} & \cdots & Z_{1n}\\ Z_{21} & Z_{22} & \cdots & Z_{2n}\\ \vdots & \vdots & \ddots & \vdots \\ Z_{m1} & Z_{m2} & \cdots & Z_{mn} \end{array}\right] \end{array}$$

where

(20) $$z_{ij}=w_{bj}\times v_{ij},\forall ij$$

Step 8: Acquiring the solutions of the positive-ideal (PI) and the negative-ideal (NI).

According to the TOPSIS method, the evaluation criteria can be divided into benefit criteria and cost criteria. Let B be a set of benefit criteria and C be a set of cost criteria. $L^{+}$ is the positive-ideal solution and $L^{-}$ represents the negative-ideal solution. Then, $L^{+}$ and $L^{-}$ can be acquired as:

(21) $$L^{+}=\left(\;\left(\underset{i}{\max}z_{\mathrm{ij}}|\;j\in B\right),\;\left(\underset{i}{\min}z_{\mathrm{ij}}|\;j\in C\right)\right)\;=\left(z_{j}^{+}|\;j=1,2,\ldots ,m\right)$$

(22) $$L^{-}=\left(\;\left(\underset{i}{\min}z_{\mathrm{ij}}|\;j\in B\right),\;\left(\underset{i}{\max}z_{\mathrm{ij}}|\;j\in C\right)\right)\;=\left(z_{j}^{-}|\;j=1,2,\ldots ,m\right)$$

Step 9: Measuring the distance from NID (solution of NI) and PID (solution of PI).

To calculate the distance from NID or PID to each alternative $L_{i}$. The Euclidean distance is expressed by the following calculation formula:

(23) $${\text{d}}^{+}=\sqrt{\overset{n}{\underset{j=1}{\sum }}{\left(z_{\mathrm{ij}}-z_{\mathrm{ij}}^{+}\right)}^{2}}$$

(24) $$d^{-}=\sqrt{\overset{n}{\underset{j=1}{\sum }}{\left(z_{\mathrm{ij}}-z_{\mathrm{ij}}^{-}\right)}^{2}}$$

Step 10: Calculating the relative proximity of PID.

The relative proximity of an alternative $L_{i}$ to the positive-ideal solution (PID) $L^{+}$ can be shown as follows:

(25) $$\tau_{i}=\frac{d^{-}}{d^{+}+d^{-}}\;,\;\mathrm{where}\;0\leq \tau_{i}\leq 1$$

Step 11: Sorting the alternatives.

According to the relative proximity of each alternative, alternatives are ranked in descending order of $\tau_{i}$’s value. Some alternatives closer to PID will have larger relative proximity values.

In the end, the most appropriate choice will be the one with the highest proximity value.

## 4. Numerical Execution Example of Building Material Supplier Selection

The venture capital company hopes to choose the most suitable supplier based on several standard conditions of the investment target. Therefore, five construction material suppliers were further selected as the evaluation of the alternatives. Refer to the information of some building materials suppliers in Taiwan and quote or modify the input values. The name of the supplier has not been disclosed to avoid unnecessary commercial disputes. The result of the hybrid multi-criteria decision-making model for selecting suppliers will not be affected in the case where the supplier’s name is represented by the code L1~L5.

The main criteria include three aspects: service quality, product satisfaction, and supply innovation capabilities.

**Figure 5:**
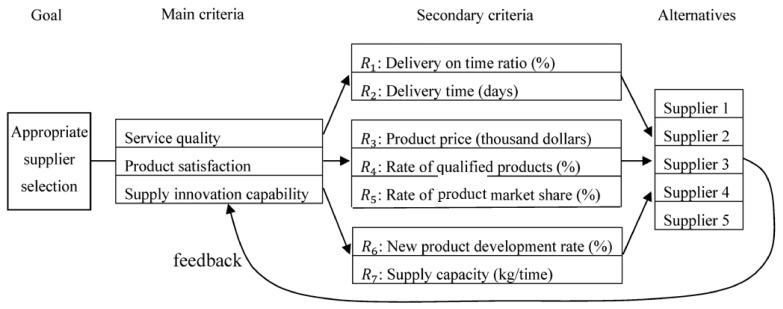
ANP analysis diagram of this study.

The secondary criteria contain three clusters. Cluster 1 contains 2 criterion, such as $R_{1}$: Delivery on time ratio (%), $R_{2}$: Delivery time (days). Cluster 2 contains 3 criterion, such as $R_{3}$: Product price (thousand dollars), $R_{4}$: Rate of qualified products (%), $R_{5}$: Rate of product market share (%). Cluster 3 contains two criterion, such as $R_{6}$: New product development rate (%), $R_{7}$: Supply capacity (kg/time). The analysis diagram of the Analytic Network Process (ANP) is shown in Figure 5.

Step 1: The establishment of the decision matrix.

A decision matrix containing seven criteria and five schemes was established. The decision matrix is expressed as follows.

$$C=\begin{array}{cc} & \begin{array}{cccc} \;R_{1}\; & R_{2}\; & \cdots \; & R_{7} \end{array}\\ \begin{array}{c} L_{1}\\ L_{2}\\ \vdots \\ L_{5} \end{array} & \left[\begin{array}{cccc} C_{11} & C_{12} & \cdots & C_{17}\\ C_{21} & C_{22} & \cdots & C_{27}\\ \vdots & \vdots & \ddots & \vdots \\ C_{51} & C_{52} & \cdots & C_{57} \end{array}\right] \end{array}=\begin{array}{cc} & \begin{array}{ccccccc} R_{1}\; & R_{2}\; & R_{3}\; & R_{4}\; & R_{5}\; & R_{6}\; & R_{7} \end{array}\\ \begin{array}{c} L_{1}\\ L_{2}\\ L_{3}\\ L_{4}\\ L_{5} \end{array} & \left[\begin{array}{ccccccc} 0.92 & 12 & 58 & 0.93 & 0.20 & 0.75 & 66\\ 0.95 & 10 & 53 & 0.97 & 0.18 & 0.71 & 61\\ 0.88 & 13 & 55 & 0.95 & 0.19 & 0.73 & 63\\ 0.93 & 11 & 56 & 0.92 & 0.17 & 0.69 & 65\\ 0.87 & 11 & 57 & 0.96 & 0.21 & 0.72 & 0.67 \end{array}\right] \end{array}$$

where C stands for criterion and A represents the alternative.

Step 2: Decision matrix normalization.

Based on the normalized performance of Formula (12) and Equtation (13), the normalized decision matrix is as follows:


$$C^{*}=\begin{array}{cc} & \begin{array}{cccc} \;V_{1}\; & V_{2}\; & \cdots \; & V_{7} \end{array}\\ \begin{array}{c} L_{1}\\ L_{2}\\ \vdots \\ L_{5} \end{array} & \left[\begin{array}{cccc} V_{11} & V_{12} & \cdots & V_{17}\\ V_{21} & V_{22} & \cdots & V_{27}\\ \vdots & \vdots & \ddots & \vdots \\ V_{51} & V_{52} & \cdots & V_{57} \end{array}\right] \end{array}=\begin{array}{cc} & \begin{array}{ccccccc} R_{1}\; & R_{2}\; & R_{3}\; & R_{4}\; & R_{5}\; & R_{6}\; & R_{7} \end{array}\\ \begin{array}{c} L_{1}\\ L_{2}\\ L_{3}\\ L_{4}\\ L_{5} \end{array} & \left[\begin{array}{ccccccc} 0.2022 & 0.2105 & 0.2079 & 0.1966 & 0.2105 & 0.2083 & 0.2050\\ 0.2088 & 0.1754 & 0.1900 & 0.2051 & 0.1895 & 0.1972 & 0.1894\\ 0.1934 & 0.2281 & 0.1971 & 0.2008 & 0.2000 & 0.2028 & 0.1957\\ 0.2044 & 0.1930 & 0.2007 & 0.1945 & 0.1789 & 0.1917 & 0.2019\\ 0.1912 & 0.1930 & 0.2043 & 0.2030 & 0.2211 & 0.2000 & 0.2081 \end{array}\right] \end{array}$$

Step 3: Using ANP method to calculate the weight of each criterion.

Assuming that specific experts and decision makers follow the steps of Section 2.2.3 ANP method, Table 3 lists the criteria weights calculated by the statistical software ‘Super Decision’ based on the ANP method to evaluate the selection of building material suppliers at each level.

The ANP weights w1pi of the main criteria can be obtained as follows:

w1pi=w1p1,w1p2,w1p3=0.3359, 0.3875,0.2766

The ANP weights w2pj of the secondary criteria can be obtained as below:

w2pi=w2p1,w2p2,w2p3=0.6518, 0.3482,0.3862, 0.2517, 0.3621,0.5371, 0.6518, 0.4629

Step 4: Using entropy method to calculate the target weight of the criterion.

According to Figure 5, the analysis clusters can be divided into four categories. Category 1 is “service quality” (2 criteria), Category 2 is “product satisfaction” (3 criteria), Category 3 is “supply innovation capability” (2 criteria), and Category 4 is “appropriate supplier selection” (7 criteria). Category 1 will be use as an example to illustrate the calculation process of entropy weight

(1) Normalizing initial data matrix.

According to the decision matrix in Step 1, set up 2 criteria and 5 alternatives to form the initial data matrix of evaluation system of Category 1.


$$C_{E1}=\begin{array}{cc} & \begin{array}{cc} \;R_{1}\; & R_{2}\; \end{array}\\ \begin{array}{c} L_{1}\\ L_{2}\\ \vdots \\ L_{5} \end{array} & \left[\begin{array}{cc} C_{11} & C_{12}\\ C_{21} & C_{22}\\ \vdots & \vdots \\ C_{51} & C_{52} \end{array}\right] \end{array}=\begin{array}{cc} & \begin{array}{cc} R_{1}\; & R_{2}\; \end{array}\\ \begin{array}{c} L_{1}\\ L_{2}\\ \vdots \\ L_{5} \end{array} & \left[\begin{array}{cc} 0.92 & 12\\ 0.95 & 10\\ 0.88 & 13\\ 0.93 & 11\\ 0.87 & 11 \end{array}\right] \end{array}$$

Because $R_{1}\;$is a benefit criterion, $R_{1}\;$‘s element normalization applies to Equation (3) $Y_{j}^{\prime }=\frac{y_{\mathrm{ij}-}\underset{i}{\min}\left\{y_{\mathrm{ij}}\right\}}{\underset{i}{\max}\left\{Y_{j}\right\}-\underset{i}{\min}\left\{Y_{j}\right\}}$. Taking $c_{11}^{\prime }$ as an example, the normalized calculation formula of $c_{11}^{\prime }$ is as follows:

$$c_{11}^{\prime }=\frac{c_{11-}\underset{1}{\min}\left\{c_{1j}\right\}}{\underset{1}{\max}\left\{c_{1j}\right\}-\underset{1}{\min}\left\{c_{1j}\right\}}=\frac{0.92-0.87}{0.95-0.87}=\langle 0.6250\rangle$$

Similarly, in order to calculate the normalized values of other elements of the $c_{E1}^{\prime }\;$matrix, we obtain the following matrix $C_{E1}^{\prime }$ as seen below:

$$C_{E1}=\begin{array}{cc} & \begin{array}{cc} \;R_{1}\; & R_{2}\; \end{array}\\ \begin{array}{c} L_{1}\\ L_{2}\\ \vdots \\ L_{5} \end{array} & \left[\begin{array}{cc} C_{11}^{\prime } & C_{12}^{\prime }\\ C_{21}^{\prime } & C_{22}^{\prime }\\ \vdots & \vdots \\ C_{51}^{\prime } & C_{52}^{\prime } \end{array}\right] \end{array}=\begin{array}{cc} & \begin{array}{cc} R_{1}\; & R_{2}\; \end{array}\\ \begin{array}{c} L_{1}\\ L_{2}\\ \vdots \\ L_{5} \end{array} & \left[\begin{array}{cc} 0.6250 & 0.3333\\ 1.0000 & 1.0000\\ 0.1250 & 0.0000\\ 0.7500 & 0.6667\\ 0.0000 & 0.6667 \end{array}\right] \end{array}$$

(2) Calculate the proportion of the j-th criterion i-th evaluation object $y_{\mathrm{ij}}^{\prime }.$.

Accordi to Equation (4) $q_{\mathrm{ij}}=\frac{y_{\mathrm{ij}}^{\prime }}{\sum_{i=1}^{m}y_{\mathrm{ij}}^{\prime }}$ and taking $q_{11}\;$as an example, the proportion calculation formula of $q_{11}$ is as follows:

$$q_{11}=\frac{c_{11}^{\prime }}{\sum_{i=1}^{5}c_{i1}^{\prime }}=\frac{0.6250}{0.6250+1.0000+0.1250+0.7500+0.0000}=0.2500$$

Similarly, in order to calculate the proportion values for other elements of the $Q_{E1}^{\prime }$ matrix, we obtain the matrix Z as seen below:

$$Q=\begin{array}{cc} & \begin{array}{cc} \;R_{1}\; & R_{2}\; \end{array}\\ \begin{array}{c} L_{1}\\ L_{2}\\ \vdots \\ L_{5} \end{array} & \left[\begin{array}{cc} Q_{11}^{\prime } & Q_{12}^{\prime }\\ Q_{21}^{\prime } & Q_{22}^{\prime }\\ \vdots & \vdots \\ Q_{51}^{\prime } & Q_{52}^{\prime } \end{array}\right] \end{array}=\begin{array}{cc} & \begin{array}{cc} R_{1}\; & R_{2}\; \end{array}\\ \begin{array}{c} L_{1}\\ L_{2}\\ \vdots \\ L_{5} \end{array} & \left[\begin{array}{cc} 0.2500 & 0.1250\\ 1.40000 & 1.3750\\ 0.0500 & 0.0000\\ 0.3000 & 0.2500\\ 0.0000 & 0.2500 \end{array}\right] \end{array}$$

(3) Calculate the value of the information entropy of the j-th criterion.

Based on Equation (6)$,{\;e}_{j}=-\frac{1}{\ln m}\sum_{i=1}^{m}q_{\mathrm{ij}}\ln q_{\mathrm{ij}}$, taking $e_{1}$ as an example, the proportion calculation formula of $e_{1}$ is as follows:

$$e_{1}=-\frac{1}{\ln5}\overset{5}{\underset{i=1}{\sum }}q_{i1}\ln q_{i1}=-\frac{1}{\ln5}(0.2500\times \ln0.2500+0.4000\times \ln0.4000+0.0500\times \ln0.0500\;+0.3000\times \ln0.3000+0.000\times \ln0.000)=0.7606$$

In the same calculation, we can obtain $e_{2}=$ 0.8207.

(4) Calculate the information utility for the j-th criterion.

Based on Equation (7) $t_{j}$ = 1 − $e_{j}$, the information utility $t_{j}$ for the j-th criterion can be calculated as follows:

$$t_{1}\;=1-e_{1}\;=1-0.7606=0.2394;\;t_{2}\;=1-e_{2}=1-0.8207=0.1793.$$

(5) Calculate the entropy weight of the j-th criterion.

According to Equation (8) $w_{\mathrm{ej}}=\frac{t_{j}}{\sum_{j=1}^{n}t_{j}}$, the entropy weight $w_{j}\;$of the j-th criterion can be calculated as follows:

$$w_{e1}=\frac{t_{1}}{\sum_{j=1}^{2}t_{j}}=\frac{0.2394}{0.2394+0.1793}=0.5718$$

$$w_{e2}=\frac{t_{2}}{\sum_{j=1}^{2}t_{j}}=\frac{0.1793}{0.2394+0.1793}=0.4282$$

In the same way, the entropy weights of the other three categories can also be obtained. Therefore, the entropy method is used to evaluate the weights of the main criteria and secondary criteria for the selection of building materials suppliers as shown in Table 4.

The entropy weight w1ei of the main criteria can be obtained as follows:

w1ei=w1e1,w1e2,w1e3=0.2878, 0.4361, 0.2761

The entropy weight w2ej of the secondary criteria can be obtained as follows:

w2ej=w2e1,w2e2,…,w2e7=0.5718, 0.4282, 0.3402, 0.3321, 0.3277, 0.5087, 0.4913

Step 5: Calculating the combination weights of the criteria.

**Figure 6:**
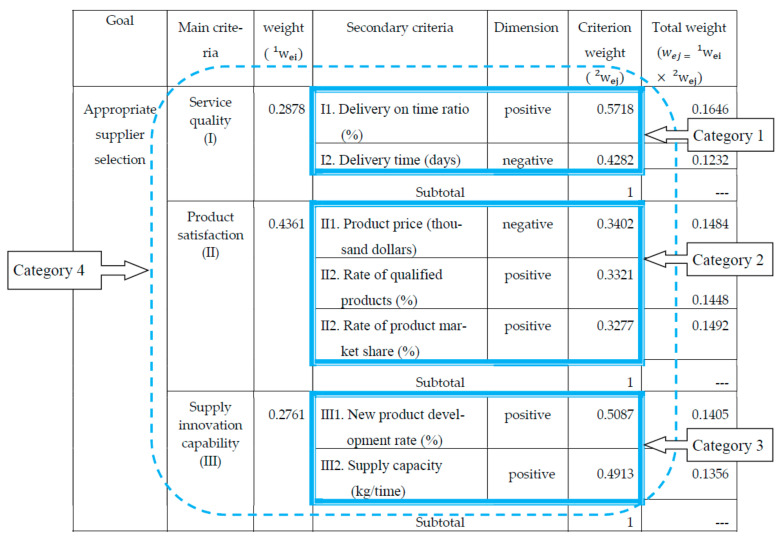
Weights of main criterion and secondary criterion of building material supplier selection evaluated with the entropy method.

**Figure 7:**
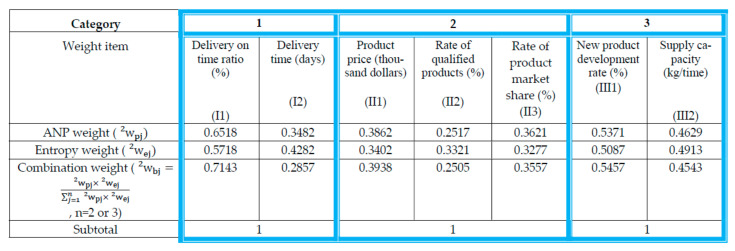
Secondary criteria weights of building materials supplier selection evaluated by the combination weighting method.

According to Equation (17) wibj=wipj×wiej∑j=1nwipj×wiej, where $w_{pj}$ is the subjective weight derived from the ANP method and $w_{ej}$ is the objective weight calculated by entropy, then use the combined weighting method to obtain the combined weight of each criterion (w1bi and w2bi), as shown in Table 4 and Figure 6 and Figure 7.

The combination weight w1bi of the main criteria can be obtained as follows:

w1bi=w1b1,w1b2,w1b3=0.2826, 0.4941,0.2233

The combined weighting method needs to be calculated individually based on the three categories, so n is equal to 2 or 3. Moreover, the sum of the weights of the individual secondary criteria of the three categories is still equal to 1 after the combined weighting method.

The combination weights w2bj of the secondary criteria can be obtained as seen below:

w2bj=w2b1,w2b2,…,w2b7=0.7143, 0.2857, 0.3938, 0.2505, 0.3557, 0.5457, 0.4543

Step 6: Establishing the ANP-entropy weight of the decision matrix.

Based on Table 4 and Figure 7, and by Equation (18), wbj =w1bi×w2bj, Table 5 showed the ANP-entropy weight ($w_{\mathrm{bj}\;}$) of the decision matrix.

We can obtain the ANP-entropy weight ($w_{bj\;}$) of each criterion by means of the equation listed below:

$$w_{bj}=\left(w_{b1},w_{b2},\ldots ,w_{b7}\right)=\left(0.2019,\;0.0807,\;0.1946,\;0.1238,\;0.1758,\;0.1219,\;0.1014\right)$$

Step 7: A combined weighted normalized decision matrix is established.

The combined weighted normalized decision matrix by Equations (19) and (20), $z_{ij}=w_{bj}\times v_{ij},$ can be expressed as:

$$Z=\begin{array}{cc} & \begin{array}{cccc} \;R_{1}\; & R_{2}\; & \cdots \; & R_{7} \end{array}\\ \begin{array}{c} L_{1}\\ L_{2}\\ \vdots \\ L_{5} \end{array} & \left[\begin{array}{cccc} V_{11} & V_{12} & \cdots & V_{17}\\ V_{21} & V_{22} & \cdots & V_{27}\\ \vdots & \vdots & \ddots & \vdots \\ V_{51} & V_{52} & \cdots & V_{57} \end{array}\right] \end{array}=\begin{array}{cc} & \begin{array}{ccccccc} R_{1}\; & R_{2}\; & R_{3}\; & R_{4}\; & R_{5}\; & R_{6}\; & R_{7} \end{array}\\ \begin{array}{c} L_{1}\\ L_{2}\\ L_{3}\\ L_{4}\\ L_{5} \end{array} & \left[\begin{array}{ccccccc} 0.0408 & 0.0170 & 0.0405 & 0.0243 & 0.0370 & 0.0254 & 0.0208\\ 0.0422 & 0.0142 & 0.0370 & 0.0254 & 0.0333 & 0.0240 & 0.0192\\ 0.0390 & 0.0184 & 0.0384 & 0.0249 & 0.0352 & 0.0247 & 0.0198\\ 0.0413 & 0.0156 & 0.0391 & 0.0241 & 0.0315 & 0.0234 & 0.0205\\ 0.0386 & 0.0156 & 0.0398 & 0.0251 & 0.0389 & 0.0244 & 0.0211 \end{array}\right] \end{array}$$

Step 8: Solve for the negative-ideal (NI) and the positive-ideal (PI).

These seven criteria are divided into cost criteria or benefit criteria. Cost criteria are C = {$C_{2}$,${\;C}_{3}$}, such as “Delivery time (days)” and “Product price (thousand dollars)”. However, benefit criteria are B={$C_{1}$,$C_{4}$,$C_{5},C_{6},C_{7}$}, such as “Delivery on time ratio (%)”, “Rate of qualified products (%)”, “Rate of product market share (%)”, “New product development rate (%)”, and “Supply capacity (kg/time)”. Then, we obtain the positive ideal (PI) and negative ideal (NI) solutions as follows:

$$L^{+}=\left\{0.0422,\;0.0142,0.0370,\;0.0254,\;0.0389,\;0.0254,\;0.0211\right\}$$

$$L^{-}=\left\{0.0386,\;0.0184,\;0.0405,\;0.0241,\;0.0315,\;0.0234,\;0.0192\right\}$$

Step 9: Calculate the Euclidean distance from NI solution (NIS) and PI solution (PIS).

According to the normalized Euclidean distance, to measure the distance between the positive and negative solutions of each alternative by Equation (23) $d^{+}=\sqrt{\sum_{j=1}^{n}{\left(z_{\mathrm{ij}}-z_{\mathrm{ij}}^{+}\right)}^{2}}$ and Equation (24) $d^{-}=\sqrt{\sum_{j=1}^{n}{\left(z_{\mathrm{ij}}-z_{\mathrm{ij}}^{-}\right)}^{2}}$. The measurement results are shown in Table 6 and Table 7.

Step 10: Calculation of the Relative Proximity of PIS.

**Figure 8:**
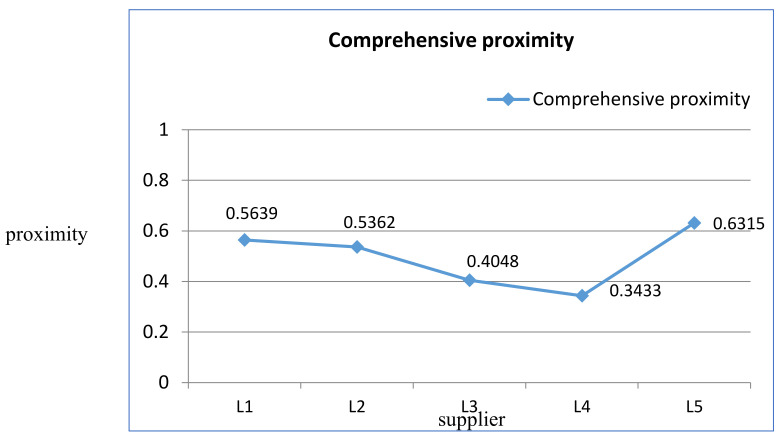
Comprehensive proximity of five supplier alternatives.

Equation (25) $\tau_{i}=\frac{d^{-}}{d^{+}+d^{-}}\;$shows the relative proximity of an alternative $L_{i}$ with regard to the positive-ideal solution (PIS). The results are shown in Table 8 and Figure 8. The closer the proximity is to 1, the higher the overall performance of the selected supplier (that is, the most suitable supplier), as shown in Figure 8.

Step 11: Sorting the Options.

After calculating the relative proximity of each alternative to the ideal solution, the solutions are arranged in descending order of $\tau_{i}$. The five options are arranged in the order of $L_{5}\;$> $L_{1\;}$> $L_{2\;}$> $L_{3\;}$> $L_{4}$, as shown in Table 9. Among the five alternatives, $L_{5}$ was selected as a suitable building material supplier.

## 5. Results and Discussion

In the final stage, the multi-criteria evaluation method must conduct a sensitivity analysis to analyze the relationship between the weight of the alternatives and the proximity of TOPSIS. In this section, we will conduct a sensitivity analysis and discuss the findings of this article. From the second stage to the fourth stage of the research framework of this article, a weight value can be obtained that can replace the subjective weight value set by the decision maker in the traditional TOPSIS method, that is, the ANP-entropy weight value of TOPSIS.

From step 6, we can obtain the value of the ANP-entropy weight vector, that is, $w_{bj}=\left(w_{b1},w_{b2},\ldots ,w_{b7}\right)=\left(0.2019,\;0.0807,\;0.1946,\;0.1238,\;0.1758,\;0.1219,\;0.1014\right)$. This means that the individual impact of each criterion on the alternatives is 20.19%, 8.07%, 19.46%, 12.38%, 17.58%, 12.19% and 10.14%. By combining the subjective weight (ANP) with the objective weight (entropy), the deviation of the subjective weight can be reduced and the status can be more truly reflected. In the third stage, the TOPSIS method is improved by using the ANP-entropy weight, and a hybrid ANP-entropy weighted TOPSIS model is established.

Based on the hybrid ANP-entropy TOPSIS model, the value of the evaluation index $\tau_{}$ represents the relative proximity of each alternative. We can arrange the alternatives in the order of $L_{5}$, $L_{1}$, $L_{2}$, $L_{3}$, and $L_{4}$ according to the value of $\tau_{}$ from high to low. Finally, the suitable supplier is determined as $L_{5}$. It can be seen from the results that the research framework proposed in this paper provides a reference value for decision makers and has the advantage of choosing a suitable solution. In order to verify the robustness and stability of the hybrid evaluation model, a systematic sensitivity analysis was carried out and compared with the ANP-based TOPSIS model.

According to Table 3 and Table 5, the main criterion and secondary criterion weights belonging to ANP-based TOPSIS and ANP-entropy TOPSIS can be shown as Table 10 and Table 11.

**Figure 9:**
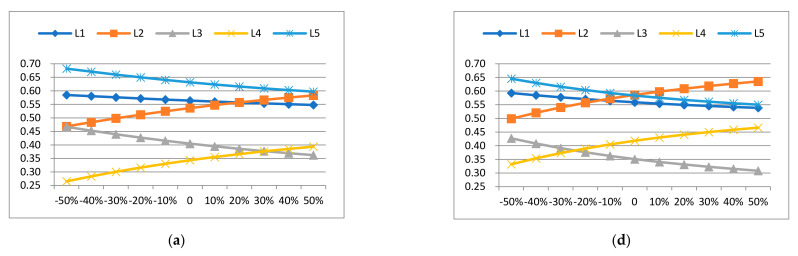

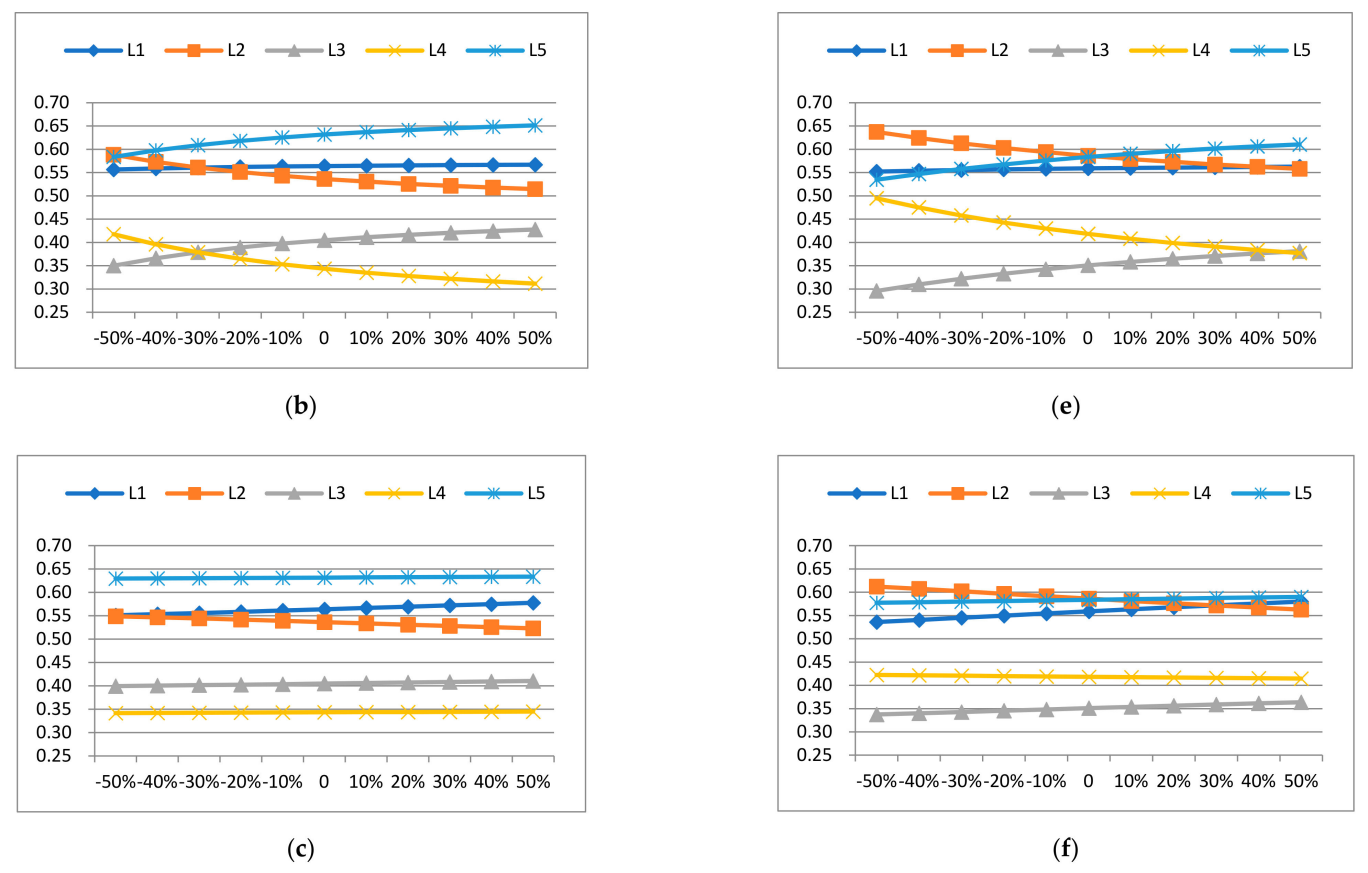
ANP-entropy TOPSIS vs. ANP-based TOPSIS—sensitivity analysis of the main criterion weight to the outcome of the alternatives. (**a**) Fluctuation of the main criterion (I) weight (w1p1inw1b1) p> to the outcome of the alternatives in ANP-entropy TOPSIS. (**b**) Fluctuation of the main criterion (II) weight (w1p2inw1b2) to the outcome of the alternatives in ANP-entropy TOPSIS. (**c**) Fluctuation of the main criterion (III) weight (w1p3inw1b3) to the outcome of the alternatives in ANP-entropy TOPSIS. (**d**) Fluctuation of the main criterion (I) weight (w1p1) to the outcome of the alternatives in ANP-based TOPSIS. (**e**) Fluctuation of the main criterion (II) weight (w1p2) to the outcome of the alternatives in ANP-based TOPSIS. (**f**) Fluctuation of the main criterion (III) weight (w1P3) to the outcome of the alternatives in ANP-based TOPSIS.

First, when the weights of the main criterion (I), (II), and (III) vary in the range of −50%, −40%, …, 40%, and 50%, explore the relative closeness of the alternatives in ANP-entropy TOPSIS and AHP-based TOPSIS. The corresponding relationship is shown in Table 12 and Figure 9. Based on the above analysis, it can be seen that the ranking of each alternative has not changed, which indicates that the value of the main criterion weight will not affect the ranking of alternatives.

No matter how the main criterion weight changes, from the point of view of the best choice, the most suitable choice is still $L_{5}$ in ANP-entropy TOPSIS.

Comparing the sensitivity analysis of the results of ANP-entropy TOPSIS (Figure 9a–c) and ANP-based TOPSIS (Figure 9d–f), we can know that ANP-entropy TOPSIS is a more stable and effective evaluation model in selecting a building material supplier than ANP-based TOPSIS.

In short, the sensitivity analysis proves that the evaluation results of the hybrid evaluation model established are reliable and valid. After sensitivity analysis of building material suppliers, the stability, feasibility, and effectiveness of the hybrid multi-criteria evaluation model for solving MCDM problems are verified.

## 6. Conclusions

This study evaluates the choice of building materials suppliers from a theoretical and practical perspective, with the purpose of establishing a new ANP-entropy weighted TOPSIS model. This article introduces how to systematically and comprehensively use ANP, entropy weight and TOPSIS methods to establish an ANP-entropy weight TOPSIS model, to achieve the purpose of this article. The research results and special advantages are as follows:

The ANP-entropy weight can replace the weight determined subjectively by the decision maker in the TOPSIS method. Integrating the entropy objective weight and the subjective weight of ANP into an eclectic weight, decision makers can evaluate potential suppliers more comprehensively and scientifically. For different levels of entropy weight and the weight of the ANP method, the calculation combination needs to be calculated separately. The total weight value (ANP-entropy weight) is a combination of the weights of each layer, and then the weights of each layer are multiplied together. The decision selection based on the ANP-entropy weight TOPSIS model has more stable, effective, and reliable results compared with the TOPSIS model based only on the AHP weight.

The main contributions of this article are shown in the following aspects:

(1) Under the condition of a suitable MCDM solution, to combine ANP-entropy weights and TOPSIS method. When the decision maker has a strong subjective consciousness and is in an environment with insufficient information, this model can provide effective information for decision making.
(2) The subjective weight directly set by the decision maker in the TOPSIS method is replaced by a compromise weight that combines subjective weight (ANP weight) and objective weight (entropy weight). In other words, the decision bias caused only by subjective and personal conscious judgments can be improved when the ANP-entropy weight replaces the subjective weight.
(3) On the basis of combining the subjective and objective weights of ANP-entropy weight, the TOPSIS method is extended. Under the new combined weight condition, construct the normalized weight matrix and calculate the relative closeness. Relative proximity can be used as the basis for the selection of suitable suppliers.

In multi-criteria decision making, the theoretical and practical application of the TOPSIS method based on ANP-entropy weights, considering the mutual influence of factors and improving the subjective opinions of decision makers, has a good application prospect. The new hybrid multi-criteria evaluation model can handle decision-related issues in multi-criteria fields, such as location selection, planning, and construction plan selection and other decision-making disciplines. The results of this research enable us to take an important step in the application of this model and be able to use the ANP-entropy weighted TOPSIS model more practically in the future.

## Figures and Tables

**Table 1:** Comparison of Analytic Network Process (ANP) and Analytic Hierarchy Process (AHP) [72].

Layer Level	ANP	AHP
Level 1	ultimate goal	ultimate goal
Level 2	cluster, component	facet
Level 3	element (criterion)	criterion
Lowest level	alternative	alternative

**Table 2:** Combined weights of each level of ANP.

		Q2-level	$Q2_{1}$	$Q2_{2}$	⋯	$Q2_{n}$	Q1-Level Element Combination Weight
	Weight						
Q1-level			$q2_{1}$	$q2_{2}$	⋯	$q2_{n}$	
$Q1_{1}$			$q1_{1}^{1}$	$q1_{1}^{2}$	⋯	$q1_{1}^{n}$	$q1_{1}=\overset{n}{\underset{i=1}{\sum }}q2_{i}q1_{1}^{i}$
$Q1_{2}$			$q1_{2}^{1}$	$q1_{2}^{2}$	⋯	$q1_{2}^{n}$	$q1_{2}=\overset{n}{\underset{i=1}{\sum }}q2_{i}q1_{2}^{i}$
⋮			⋮	⋮		⋮	⋮
$Q1_{m}$			$q1_{m}^{1}$	$q1_{m}^{2}$	⋯	$q1_{m}^{n}$	$q1_{m}=\overset{n}{\underset{i=1}{\sum }}q2_{i}q1_{m}^{i}$

**Table 3:** Weights of main criterion and secondary criterion of building material supplier selection evaluated with ANP method.

Goal	Main Criteria	Weight w1pi	Secondary Criteria	Dimension	Criterion Weight w2pj	Total Weight (wpj=w1pi×w2pj)
Appropriate Supplier Selection	Service quality (I)	0.3359	I1. Delivery on time ratio (%)	positive	0.6518	0.2189
			I2. Delivery time (days)	negative	0.3482	0.1170
			Subtotal		1	---
	Product satisfaction (II)	0.3875	II1. Product price (thousand dollars)	negative	0.3862	0.1497
			II2. Rate of qualified products (%)	positive	0.2517	0.0975
			II2. Rate of product market share (%)	positive	0.3621	0.1403
			Subtotal		1	---
	Supply Innovation Capability (III)	0.2766	III1. New product development rate (%)	positive	0.5371	0.1486
			III2. Supply capacity (kg/time)	positive	0.4629	0.1280
			Subtotal		1	---

**Table 4:** Main criteria weights of building material supplier selection evaluated with the combination weighting method.

Weight Item	Service Quality (I)	Product Satisfaction (II)	Supply Innovation Capability (III)
ANP weight (w1pi)	0.3359	0.3875	0.2766
Entropy weight (w1ei)	0.2878	0.4361	0.2761
Combination weight (w1bi=w1pi×w1ei∑i=13w1pi×w1ei)	0.2826	0.4941	0.2233
Subtotal	1		

**Table 5:** The ANP-entropy weight (w_bj_) calculated by the combination weighting method.

Goal	Main Criteria	Weight w1bi	Secondary Criteria	Dimension	Criterion Weight w2bjw2pj	Total weight (ANP-Entropy wbj=w1bi×w2bj)
Appropriate Supplier Selection	Service quality (I)	0.2826	I1. Delivery on time ratio (%)	positive	0.7143	0.2019
			I2. Delivery time (days)	negative	0.2857	0.0807
			Subtotal		1	---
	Product satisfaction (II)	0.4941	II1. Product price (thousand dollars)	negative	0.3938	0.1946
			II2. Rate of qualified products (%)	positive	0.2505	0.1238
			II2. Rate of product market share (%)	positive	0.3557	0.1758
			Subtotal		1	---
	Supply innovation capability (III)	0.2233	III1. New product development rate (%)	positive	0.5457	0.1219
			III2. Supply capacity (kg/time)	positive	0.4543	0.1014
			Subtotal		1	---

**Table 6:** Euclidean distance measures from the negative-ideal solution (NIS).

Alternatives	$L_{1}$	$L_{2}$	$L_{3}$	$L_{4}$	$L_{5}$
$d^{-}$	0.0067	0.0070	0.0046	0.0043	0.0083

**Table 7:** Euclidean distance measures from the positive-ideal solution (PIS).

Alternatives	$L_{1}$	$L_{2}$	$L_{3}$	$L_{4}$	$L_{5}$
Alternatives	$L_{1}$	$L_{2}$	$L_{3}$	$L_{4}$	$L_{5}$
$d^{+}$	0.0052	0.0060	0.0068	0.0083	0.0048

**Table 8:** Relative proximity of the alternatives.

Alternatives	$L_{1}$	$L_{2}$	$L_{3}$	$L_{4}$	$L_{5}$
$\tau_{i}$	0.5639	0.5362	0.4048	0.3433	0.6315

**Table 9:** The order of options.

Options	$L_{1}$	$L_{2}$	$L_{3}$	$L_{4}$	$L_{5}$
**Rank**	2	3	4	5	1

**Table 10:** Main criterion weights of ANP-based technique for order preference by similarity to an ideal solution (TOPSIS) and ANP-entropy TOPSIS.

MCDM Method	Service Quality (I)	Product Satisfaction (II)	Supply Innovation Capability (III)
ANP-based TOPSIS	0.3359	0.3875	0.2766
ANP-Entropy TOPSIS	0.2826	0.4941	0.2233

**Table 11:** Secondary criterion weights of ANP-based TOPSIS and ANP-entropy TOPSIS.

MCDM Method	Delivery on Time Ratio (%) (I1)	Delivery Time (Days) (I2)	Product Price (Thousand Dollars) (II1)	Rate of Qualified Products (%) (II2)	Rate of Product Market Share (%) (II3)	New Product Development Rate (%) (III1)	Supply Capacity (kg/time) (III2)
ANP-based TOPSIS	0.6518	0.3482	0.3862	0.2517	0.3621	0.5371	0.4629
ANP-Entropy TOPSIS	0.7143	0.2857	0.3938	0.2505	0.3557	0.5457	0.4543

**Table 12:** Sensitivity analysis of the main criterion (I) weight (w1p1inw1b1)  to the outcome of the alternatives in ANP-entropy TOPSIS.

	w1p1=−50%	w1p1= −40%	w1p1= −30%	w1p1=−20%	w1p1= −10%	w1p1=0	w1p1=10%	w1p1= 20%	w1p1= 30%	w1p1= 40%	w1p1= 50%
$L_{1}$	0.5847	0.5803	0.5760	0.5717	0.5677	0.5638	0.5602	0.5568	0.5536	0.5506	0.5478
$L_{2}$	0.4686	0.4837	0.4982	0.5118	0.5245	0.5362	0.5471	0.5571	0.5664	0.5750	0.5829
$L_{3}$	0.4669	0.4530	0.4396	0.4271	0.4155	0.4047	0.3948	0.3857	0.3773	0.3695	0.3623
$L_{4}$	0.2654	0.2837	0.3006	0.3162	0.3304	0.3434	0.3553	0.3662	0.3762	0.3854	0.3939
$L_{5}$	0.6821	0.6708	0.6600	0.6498	0.6403	0.6315	0.6234	0.6159	0.6091	0.6027	0.5969
